# Fibroblast Proliferation and Migration in Wound Healing by Phytochemicals: Evidence for a Novel Synergic Outcome

**DOI:** 10.7150/ijms.43986

**Published:** 2020-04-07

**Authors:** Roberta Addis, Sara Cruciani, Sara Santaniello, Emanuela Bellu, Giorgia Sarais, Carlo Ventura, Margherita Maioli, Giorgio Pintore

**Affiliations:** 1 Department of Chemistry and Pharmacy, University of Sassari, Via F. Muroni 23/b, 07100, Sassari, Italy;; 2 Department of Biomedical Sciences, University of Sassari, Viale San Pietro 43/B, 07100 Sassari, Italy;; 3 Department of Life and Environmental Sciences, University of Cagliari, Via Ospedale 72, 09124 Cagliari, Italy;; 4 Laboratory of Molecular Biology and Stem Cell Engineering, National Institute of Biostructures and Biosystems - Eldor Lab, Innovation Accelerator, Consiglio Nazionale delle Ricerche, Bologna, Italy.; 5 Center for Developmental Biology and Reprogramming (CEDEBIOR), Department of Biomedical Sciences, University of Sassari, Viale San Pietro 43/B, 07100 Sassari, Italy; 6 Istituto di Ricerca Genetica e Biomedica, Consiglio Nazionale delle Ricerche (CNR), Monserrato, 09042 Cagliari, Italy

**Keywords:** cellular mechanisms, cell proliferation, tissue regeneration, antioxidants, natural molecules, oxidative stress

## Abstract

Wound-healing is a dynamic skin reparative process that results in a sequence of events, including inflammation, proliferation, and migration of different cell types as fibroblasts. Fibroblasts play a crucial role in repairing processes, from the late inflammatory phase until the fully final epithelization of the injured tissue. Within this context, identifying tools able to implement cell proliferation and migration could improve tissue regeneration. Recently, plants species from all over the world are coming out as novel tools for therapeutic applications thanks to their phytochemicals, which have antioxidant properties and can promote wound healing. In this paper, we aimed at investigating antioxidant activity of waste extracts from different medicinal plants, endemic of the Mediterranean area, on fibroblast proliferation and wound healing. We determined the amount of total phenols and anti-oxidant activity by ABTS assay. We then evaluated the cytotoxicity of the compounds and the proliferative capabilities of fibroblasts by scratch assay. Our results showed that waste extracts retain antioxidant and regenerative properties, inducing tissue re-establishment after environmental stress exposure. Taken together, our findings suggest that waste material could be used in the future also in combinations to stimulate wound healing processes and antioxidant responses in damaged skin.

## Introduction

Wound-healing is a dynamic process, which, following skin injury, arises through a sequence of events, including inflammation, proliferation, and migration of different cell types as fibroblasts 1, 2. Fibroblasts play a crucial role in tissue repair, from the late inflammatory phase until the fully final epithelization of the injured tissue, by secreting growth factors, cytokines, collagens and others extracellular matrix (ECM) components 3-6. At the same time, fibroblast migration and proliferation bear crucial roles in the healing process, by initiating the proliferative phase of repair 7, 8.

Bioactive molecules found in different plant species from all over the world are now emerging as novel tools for therapeutic applications. Several studies revealed that plants produce potent antioxidants to control the oxidative stress caused by sunlight and oxygen, thus representing a source of new compounds with antioxidant activity 9. Asteraceae (Compositae), is the family with the larger number of species among the Dicotyledons. *Calendula arvensis* L. and *Helichrysum italicum (Roth) Don subsp. microphyllum (Willd.) Nym.* belongs to this family 10-12 and are known to contain saponins, triterpenic alcohols and their fatty acid esters, carotenoids, flavonoids, coumarins, essential oils, hydrocarbons and fatty acids 13. Traditional and folk medicinal uses include treatment of skin problems, fevers, chronic infections, wounds, bites and stings 14, 15. *Lavandula stoechas* L. is an important member of Labiatae (Lamiaceae) family. It is commonly used for various diseases of central nervous system (epilepsy and migraine), wound healing, decrease of blood sugar levels 16, and as antispasmodic, antiseptic, antimicrobial, sedative, diuretic and analgesic agent 17-19. Nevertheless, it is also described that Lavender has a positive effects on urinary infections, cardiac diseases and eczema 20.

Plants are rich source of phytochemicals, which can have antioxidant properties and can promote wound healing. Reactive oxygen species (ROS) at high concentration counteract wound healing processes, due to cellular membranes damages. Moreover, high levels of ROS can induce severe tissue injuries even leading to neoplastic transformation 21-23. Despite these toxic effects, ROS mediated signaling, representing a first response to different kind of cellular stressors, is also involved in tissue regeneration and fibroblast activation 24.

The biological potential of waste materials has been previously studied, in the attempt to employ post-distillation waste material of aromatic plants, thus creating both economic and ecologic benefits 25-27. Actually, after volatile oils removal, waste plant materials are still abundant in phenolic compounds, oligomers and flavonoid glycosides with various biological activities 28, therefore these compounds could still have an application 29, 30. Nevertheless, herb-herb combination have been used for thousand years, but scientific evidence of therapeutic benefits and a potential synergic effect of different compounds is not well defined yet 31.

In the present study we aimed at investigating fibroblast proliferation and wound-healing capabilities *in vitro* in the presence of waste extracts, after distillation of essential oils from different medicinal plants, endemic of the Mediterranean area. In particular, *Calendula arvensis L.*, *Lavandula stoechas* L.*, Helichrysum italicum (Roth) Don subsp. microphyllum (Willd.) Nym.* waste extracts, were tested, alone or in combination, in order to evaluate their synergic effects, for a potential antioxidant and wound healing activity.

## Materials and Methods

### Materials

Dulbecco's modified Eagle's medium (DMEM), fetal bovine serum (FBS), L-glutamine and trypsin/ EDTA were all purchased from Invitrogen, Carlsbad, CA. 200 U/mL penicillin-0.1 mg/mL streptomycin and Dulbecco's phosphate buffered saline (DPBS) were from Euroclone, Milano, Italy. Chemicals used were purchased from Sigma-Aldrich Italy (IT), and were of the highest analytical grade. Standards of chlorogenic acid, quercetin, luteolin, rosmarinic acid, apigenin, and naringenin were purchased from Extrasinthese (Lyon, France). Acetonitrile (Sigma, Milano - Italy) was of HPLC grade. Orthophosphoric acid (ACS ISO, for analysis, 85%) were purchased from Carlo Erba. Water was distilled and filtered through a Milli-Qapparatus (Millipore, Milan, Italy) before use.

### Sample collection

Plant materials, *C. arvensis, L. stoechas, H. italicum*, were collected during spring 2017 in the Asinara National Park, exactly on Cala Reale bay. The leaves were dried at room temperature for 5 days and then were cut in fragments of approximately 5 cm. Thereafter 175 gr of each fraction was placed in a round-bottom flask with 1000 ml of distilled and deionized water at final concentration of 17.5%. The flask was coupled to a Clevenger-type system to perform steam distillation. In this condition plant materials remained in contact with boiling water for 4 h in order to extract the essential oil of *C. arvensis* (C), *L. stoechas* (L), *H. italicum* (H). In order to determine the concentration of each extract, 1 mL of waste extract was lyophilized on a Eppendorf Concentrator plus, obtaining a concentration of 41.93 µg/µL for *Calendula arvensis L.*, 26.17 µg/µL for *Helichrysum italicum (Roth) Don subsp. microphyllum (Willd.) Nym.* and 39.93 µg/µL for *Lavandula stoechas L.* The residual water decoction (waste extracts), which was typically discarded, were centrifuged, filtrated and used for the assays. In addition, to evaluate the synergic effect, 500 ml of each waste extract were combined as follow:

1) *C. arvensis, L. stoechas, H. italicum (CLH)*

2) *C. arvensis, L. stoechas (CL)*

3) *C. arvensis, H. italicum (CH)*

4) *L. stoechas, H. italicum (LH)*

The final concentration of combined extracts was 38.90 µg/µL for CLH, 45.32 µg/µL for CL, 33.25 µg/µL for CH and 38.13 µg/µL for LH.

### Determination of total phenols

Total phenols were quantified by a colorimetric assay based on procedures described by Lizcano et al (2010), as previously described 32. Basically,100 µl of different concentrations of samples (from 0.1, to 1000 µg/mL) were added to 900 µl of bi-distillated water and 75 µl of Folin-Ciocalteau phenol reagent. After 2 min, 200 µl of sodium bicarbonate (75 g/l) were added to the mixture and incubated in the dark for 60 min at room temperature. The absorbance was readed at 770 nm using a 1 cm quartz cuvette on an Ultrospec 4300 pro UV-vis spectrophotometer, equipped with a temperature controller set to 25 °C. Gallic acid (1-200 µg) was used as standard. Results were expressed as µg of gallic acid equivalent (GAE) per mg of the dried plant part.

### Stock standard solutions of polyphenols

Stock standard solutions of 1000 mg/l for individual standards was prepared in methanol by dissolving an exactly known mass of solute in a specific volume of solvent. These starting solutions were used to prepare one standard mixture. A serial dilutions of this intermediary standard solution, were made by diluting more concentrated solution with 0.22M phosphoric acid to obtain mixed reference solutions in the range of 0.1-20 mg/l. These solutions were used for external standard calibration, linearity check and determination of limits of detection (LOD) and limits of quantitation (LOQ). All standard solutions were stored in the dark at -20 °C until usage.

### HPLC analysis

The HPLC experiments were performed according to a method previously reported by Sarais et al. and appropriately modified 33. Briefly, the analysis was performed on an Agilent 1100 series HPLC system (Agilent Technologies, Milan, Italy) equipped with a quaternary pump, a vacuum degasser, an autosampler and a thermostated column compartment and coupled with a diode array detector (DAD) UV6000LP (Thermo Finnigan, Milan, Italy) using a data acquisition software ChromQuest version 4.0. A reversed-phase column Kinetex C18, 100 A (150 x 4.6 mm, 5u) from Phenomenex, maintained at 22°C, was used. The chromatographic separation was obtained by gradient elution with an aqueous solution containing 0.22M phosphoric acid (solvent A) and acetonitrile (solvent B) at a constant flow rate of 0.4 ml/min. An increasing linear gradient of solvent B was used starting from 5% and reaching 80% in 120 minutes. The column was equilibrated for 15min before the next sample injections. The injection volume was 10 μl. A multi-wavelength endpoint detection was chosen for qualitative and quantitative analysis. Typical wavelength is 280 nm and 360 nm for measurement of chlorogenic acid, quercetin, luteolin, rosmarinic acid, apigenin, and naringenin respectively. The contents of the active ingredients were expressed as milligrams of active ingredient per L of extract. All analyses were replicated three times. Data were expressed as the mean ± standard deviation (SD).

### Antioxidant Assay

The ABTS (2,2-azinobis (3-ethylbenzothiazoline-6-sulfonic acid)) free radical-scavenging activity of each sample was determined according to the method described by Petretto et al (2015) 34. It involves the direct production of the blue/green ABTS·+ chromophore through the reaction between ABTS and potassium persulfate, as previously described 32. ABTS was dissolved in water to give a final concentration of 7 mM. ABTS·^+^ was produced by the reaction of ABTS stock solution with 2.45 mM potassium persulfate (final concentration) and allowing the mixture to stand in the dark at room temperature for 12-16 h before use. The ABTS·^+^ solution was diluted with bi-distillated water to an absorbance of 0.7 (±0.02) at 734 nm. In order to measure the antioxidant activity of waste extracts and their synergic effects, 100 µl of aqueous extracts solution at various concentrations (from 0.1 to 100 µg/mL) were added to 900 µL of diluted ABTS·^+^ and the absorbance recorded at time zero and after 50 min at which point the absorbance was stable. Each concentration was analyzed in triplicate. The discoloration percentage at 734 nm was calculated for each point; the antioxidant capacity of the test compound was expressed in percent inhibition (%), and the IC_50_ value, the amount of an extract that neutralize 50% of the radical, was calculated from regression analysis and expressed as mean ± SD.

### Cell Culture

The human foreskin fibroblasts 1 (HFF1), purchased from American Type Culture Collection (ATCC), were routinely cultured in DMEM low glucose supplemented with 2 mM L-glutamine and 10% (v/v) FBS. All cells were grown in 1% antibiotic/antimitotic media at 37 °C and 5% (v/v) CO_2._ Cells for the experiments were used at passage 5.

### MTT Viability assay

To evaluate cytotoxicity of different plants extracts, fibroblasts were seeded at a concentration of 7,000 cells/well and allowed to incubate overnight. Once attached at wells surface, cells were washed in phosphate buffered saline (PBS). Than cells were cultured with a medium conditioned with different concentrations (1, 5, 10 µL/mL) of each waste extracts of C, or L or H, or with the combination of 2 or 3 plants waste extracts (CLH, CL, CH, LH), for 24h, 48h, 72h, 96h or 120 hours. At the indicated time points, the culturing medium was discarded, and MTT reagent was added at the final concentration of 650 µg/mL. After 2h of incubation with 650 µg/mL MTT reagent, formazan was dissolved in DMSO and absorbance detected at 570 nm using Varian50 MPR, Microplate reader. The results expressed in OD units as compared to untreated cells. Data are expressed as mean± SD referring to the control.The viability of treated cells vs control was calculated as follow:

% cell viability = (OD570 treated cells) × 100 / (OD570 control).

### Proliferation and migration assay

Fibroblasts were seeded at a concentration of 35,000 cells/well and allowed to incubate until confluence. A scratch was made in each well using a 200μl pipette tip. Media was removed and cells were washed in PBS before adding the medium conditioned with different concentrations (1, 5, or 10 µL/mL) of each waste extracts of C, or L, or H, or with the combination of 2 or 3 plants waste extracts (CLH, CL, CH, LH).

Five different areas along the scratches of each wells were analyzed by optical microscopy after 0, 24, 48, 72, and 96 hours following the induced damage. The distance between each edges of the scratch was measured using the software ImageJ and expressed as percentage of closure of the area as compared to control untreated cells.

### Immunostaining

Fibroblasts were seeded at a concentration of 20,000 cells/well and a scratch was made in each well using a 200μl pipette tip before adding the medium conditioned with different concentrations (1, 5, or 10 µL/mL) of each waste extracts of C, or L, or H, or with the combination of 2 or 3 plants waste extracts (CLH, CL, CH, LH). Control cells were maintained in the presence of a basic growing medium. At the end of wound closure (72 hours), cells were fixed with 4% of pharaformaldeide (Sigma Aldrich Chemie GmbH, Germany) for 30 min. After permeabilization by 0.1% Triton X-100 (Life Technologies, USA)-PBS, cells were washed in PBS three times for 5 min and incubated with 3% Bovine Serum Albumine (BSA)—0.1% Triton X-100 in PBS (Life Technologies, USA) for 30 min and then exposed overnight at 4 °C to the primary anti-rabbit anti-Collagen I antibody (Abcam, United Kingdom) and anti-Collagen III antibody (Abcam, United Kingdom). Finally, cells were washed in PBS two times for 5 min and stained at 37 °C for 1 h in the dark with the fluorescence-conjugated goat anti rabbit IgG secondary antibody (Life Technologies, USA). Nuclei were labelled with 1 µg/mL 4,6-diamidino-2-phenylindole (DAPI). All microscopy analyses were performed with a confocal microscope (TCS SP5, Leica, Nussloch, Germany).

### Statistical analyses

The experiments were performed two times with three technical replicates for each treatment. All statistical analyses were performed by comparing waste extracts from leaves from C, or L or H using unpaired Student's t-test, and, using SigmaStat v 3.5 software when the data followed a normal distribution. The distribution of the sample was evaluated by the Kolmogorov-Smirnov and Shapiro tests. Differences in total phenols amounts between waste extracts of C, or L or H were assessed using linear regression in which the slope and intercept variations were compared with a global test of coincidence using GraphPad Prism 6 software. The association between variables was analyzed by the Pearson product moment correlation coefficient when the data were normally distributed. A P≤0.05 was considered statistically significant.

## Results

### Phenols are present in all waste extracts

**Figure 1:**
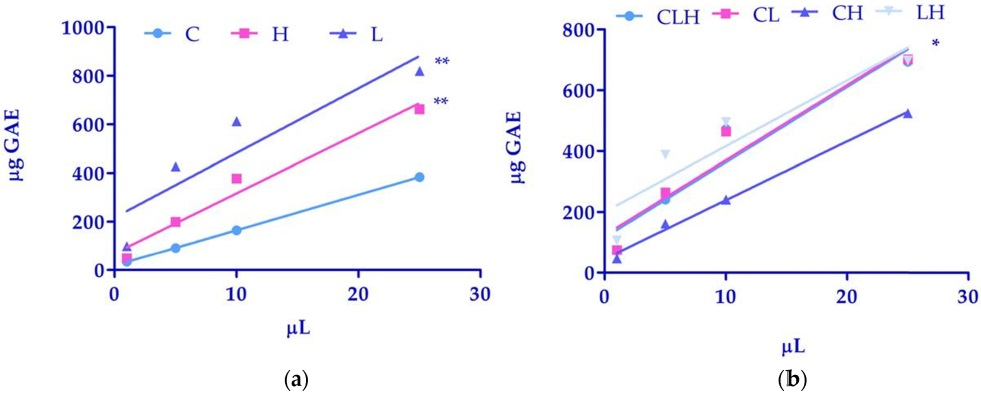
** (a)** Linear regression analysis of phenols amounts of waste extracts of *C. arvensis* (C), *L. stoechas* (L)*,* and* H. italicum* (H) and **(b)** pooled waste extracts of *C. arvensis*, *L. stoechas,* and* H. italicum* (CLH, CL, CH, LH) over volumes*.* Data were expressed as mean of 3 independent experiments (*P<0.05; **P<0.01).

A preliminary screening of polyphenol total content was performed using the Folin-Ciocalteau method. As reported in Table 1, total phenolic amounts, expressed as GAE were higher in extracts from L and H as compared to C. If we considered the differences between linear regression, the comparison of slopes, in C (14.60 ± 0.06) (light blue line), in H (24.74 ± 3.11) (pink line) and in L (26.55 ± 8.49) (purple line) were not significantly different. However, the comparison of intercepts between C (18.86 ± 0.76), H (68.98 ± 42.60) and L (217.4 ± 116.30) was significantly different with a P<0.01 (Figure 1, panel a).

Table 3 shows that the pool of CLH, CL and LH exhibited a higher phenolic amount as compared to CH. As reported in figure 1 (panel b), the comparison of slopes, in CLH (24.74 ± 5.22) (light blue line), in CL (24.63 ± 4.91) (pink line), CH (19.32 ± 1.07) (purple line), and in LH (21.60 ± 6.46) (grey line) were not significantly different. However, the comparison of intercepts between CLH (116.2 ± 71.50), CL (124.2 ± 67.31), CH (45.81 ± 14.72) and in LH (200.4 ± 88.54) was significantly different with a P<0.05 (Figure 1 panel b).

Quantitative results are reported in Table 2 and Table 4. Results are expressed as mean ± standard deviation of 3 determinations. To facilitate the qualitative methodology approach, samples were subjected to the analysis with a screening process by selecting multiple wavelengths in a diode array detector. Particularly, wavelengths of 280, and 360 were chosen to analyze phenolic fraction of samples. All phenols, identified according to their retention time and their UV spectrum, were quantified by external standard method. All waste extracts showed high quantity of water-soluble phenols belonging to different chemical classes. Due to the complexity of matrices, quantitative analysis was carried out by collecting phytochemicals in different groups according to their UV spectrum. As we can see (Table 3) caffeoylquinic acid derivatives were the most abundant compounds present in C and H (112.21 ± 6.22 mg/L and 379.76 ± 11.25 mg/L respectively). Rosmarinic acid and rosmarinic acid derivatives were higher in L (382.46 ± 15.02mg/L, 249.80 ± 8.15mg/L respectively) followed by caffeoylquinic acid derivatives (77.77 ± 5.20mg/L). Table 4 (DA FARE) shows quantitative results of pool of CLH, CL, CH and LH. Caffeoylquinic acid derivatives were the most abundant compounds present in CLH (54.84 ± 2.29 mg/L), CH (262.18 ± 9.51mg/L) and LH (207.85 ± 7.51mg/L) while rosmarinic acid was higher in CL (160.02 ± 8.98mg/L).

### Plant waste extracts exhibit antioxidant properties related to the phenolic concentration

**Figure 2:**
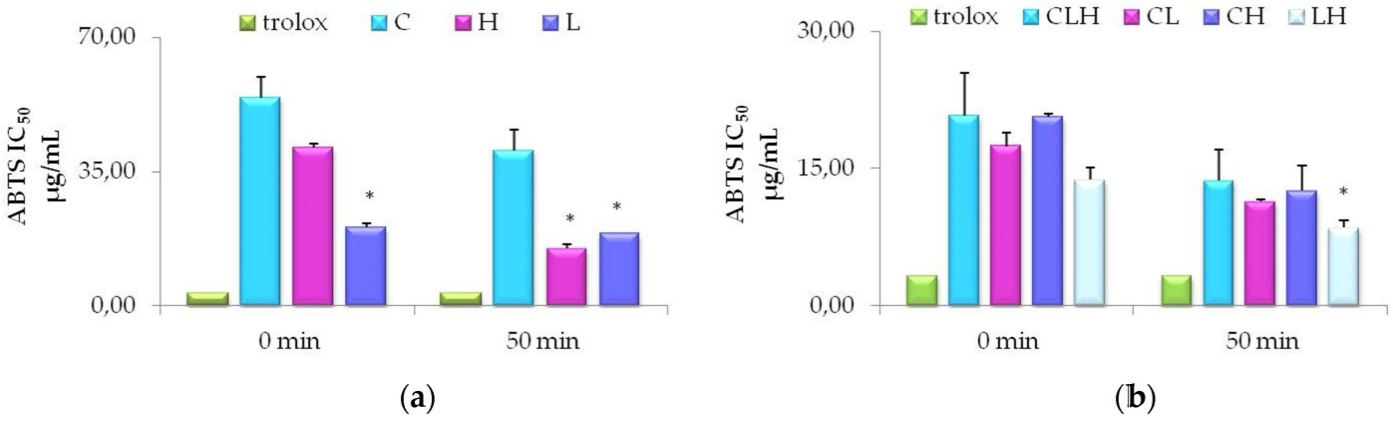
Time-course of antioxidant activity of waste extracts from leaves of *C. arvensis* (C), *L. stoechas* (L)*,* and* H. italicum* (H) and pooled waste extracts (CLH, CL, CH, LH) evaluated by ABTS assays. **(a)** Free radical scavenging of 50% of ABTS by Trolox, used as standard, C or L or H at the indicated time-points. The values are expressed as mean ± SD of three independent assays (*P < 0.05). **(b)** Free radical scavenging of 50% of ABTS by Trolox, and pooled waste extracts CLH, CL, CH, LH at the indicated time points. The values are expressed as mean ± SD of three independent assays (*P < 0.05).

As shown in Figure 2 (panel a), antioxidant activity of L extract (purple bars) was significantly higher (P<0.01) as compared to C extract (light blue bars), along with the ABTS assay-time course. Also, H (pink bars) showed a high antioxidant activity similar to L after 50 min of incubation, but slightly lower if compared to C, at time zero*.* This data is related to the minor concentration of total phenolic amounts in C (Table 1). We also show (Table 5) a positive Spearman correlation between antioxidant activity measured by ABTS and total phenolic contents (P<0.05). Pooled waste extracts (CLH, CL, CH, LH) exhibited a sensible antioxidant activity, nearly superimposable to the antioxidant Trolox (green bars), used as positive control. The higher antioxidant activity was obtained with LH (IC50 of 8.54 ± 0.82 µg/mL), showing an effect similar to Trolox. If we consider the difference between groups of pooled waste extracts, antioxidant activity was significantly higher, after 50 min, in LH (grey bars) as compared to CL (pink bars) (figure 2 panel b).

### Waste extracts stimulated cell viability

**Figure 3:**
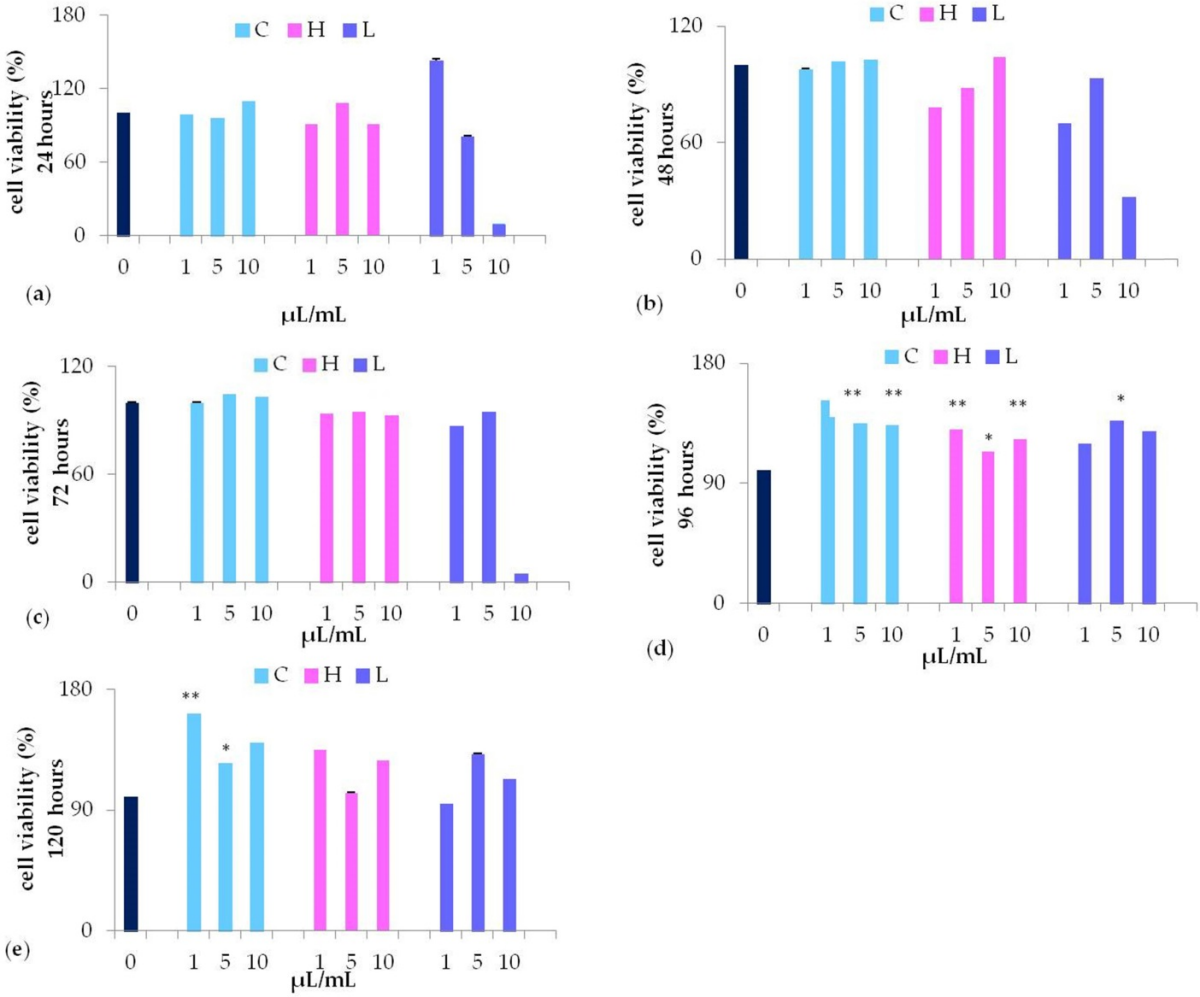
MTT assay of waste extract of *C. arvensis* (C)*, L. stoechas* (L)*,* and* H. italicum* (H) at different concentration (1, 5, 10 μL/mL) after 24h** (a)**, 48h **(b)**, 72h **(c)**, 96h** (d)** and 120h **(e)** (* p<0.05 ** p<0.01 as compared to untreated control (0).

MTT assay revealed that 10 µL/mL L extract (purple bars) significantly decreased cell viability, while stimulating cell viability at lower concentrations (5, or 1 µL/mL) (Figure 3, panel a). Nevertheless, only after 96 hours of culturing with 5 µL/mL L waste extract the stimulation was statistically different (P<0.05) as compared to control untreated cells (Figure 3, panel d). No cytotoxic effects were detected when cells were cultured with different concentrations (from 1 to 10 µL/mL) of C waste extract (light blue bars). However, in cells cultured with C waste extract cell viability was significantly induced after 96-120 hours of culturing, as compared to control untreated cells, being more effective at a lower concentration (1 µL/mL) (Figure 3, panel d-e). A similar effect on cell viability could be detected when cells were cultured for 96 hours in the presence of 1-10 μL/mL H (pink bars)* (*P <0.01 and P <0.05 respectively) waste extract (Figure 3, panel d).

**Figure 4:**
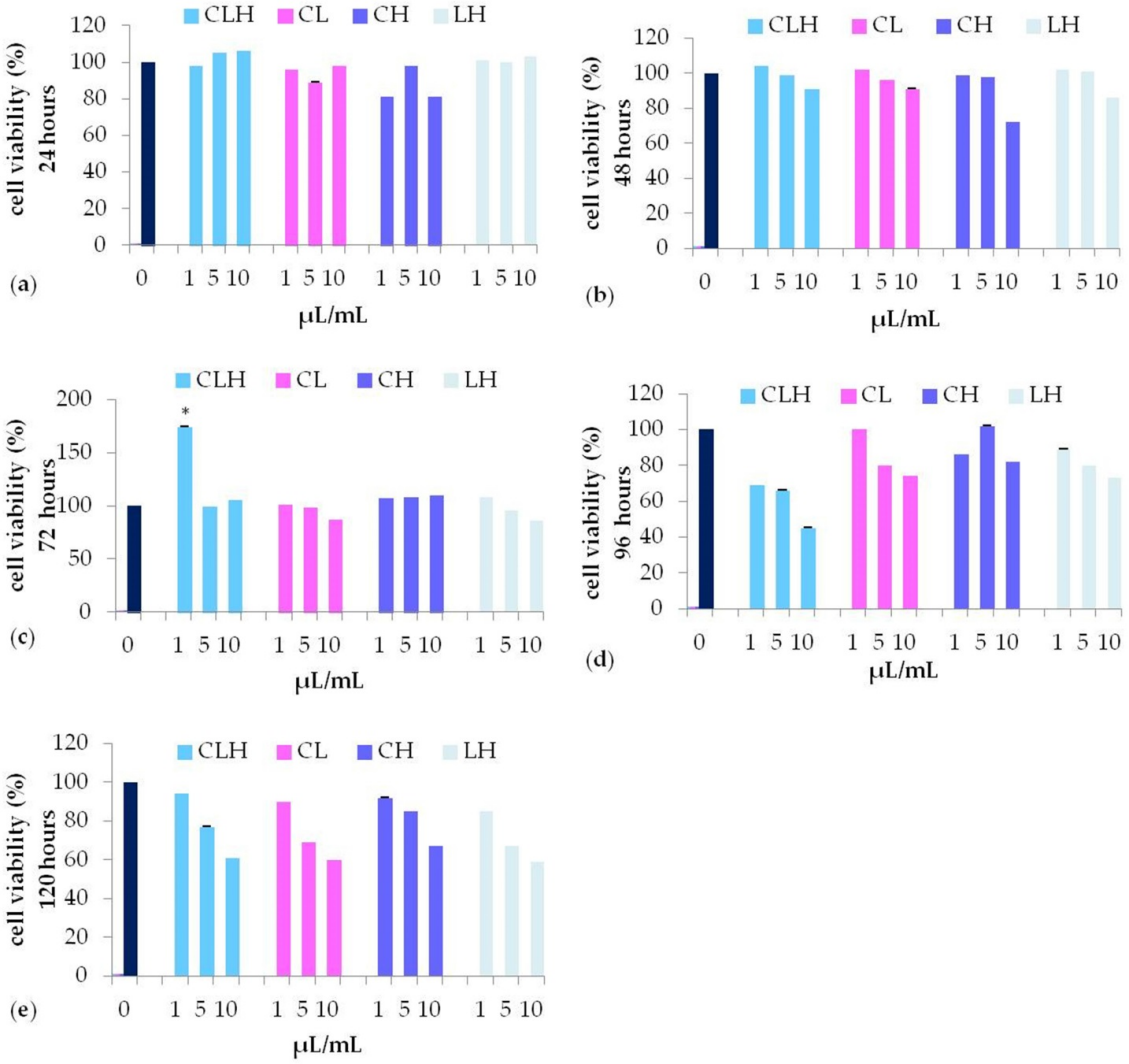
MTT assay of different concentration (1, 5, 10 μL/mL) of pooled waste extracts of *C. arvensis, L. stoechas,* and* H. italicum* CLH (light blue bar), CL (pink bar), CH (purple bar), LH (grey bar), after 24h **(a)**, 48h** (b)**, 72h **(c)**, 96h **(d)** and 120h **(e)** (* p<0.05 as compared to untreated control (0).

In the presence of all the pooled waste extracts, CLH (light blue bars), CL (pink bars), CH (purple bars), LH (grey bars) (Figure 4) , cell viability was similar to control untreated cells until 72 h of treatment, while decreasing for longer periods (96, or 120 hours) (Figure 4, panel d-e) for all the concentration tested. In particular, CLH pooled waste extracts (light blue bars), were able to induce fibroblast viability with a trend comparable to control untreated cells (dark blue bars) (Figure 4, panel a-e). Nevertheless, only after 72 h of culturing with 1 µL/mL CLH, cell viability was significantly higher (P<0.05) as compared to control untreated cells (Figure 4, panel c).

### Waste extracts were able to induce wound repair

**Figure 5:**
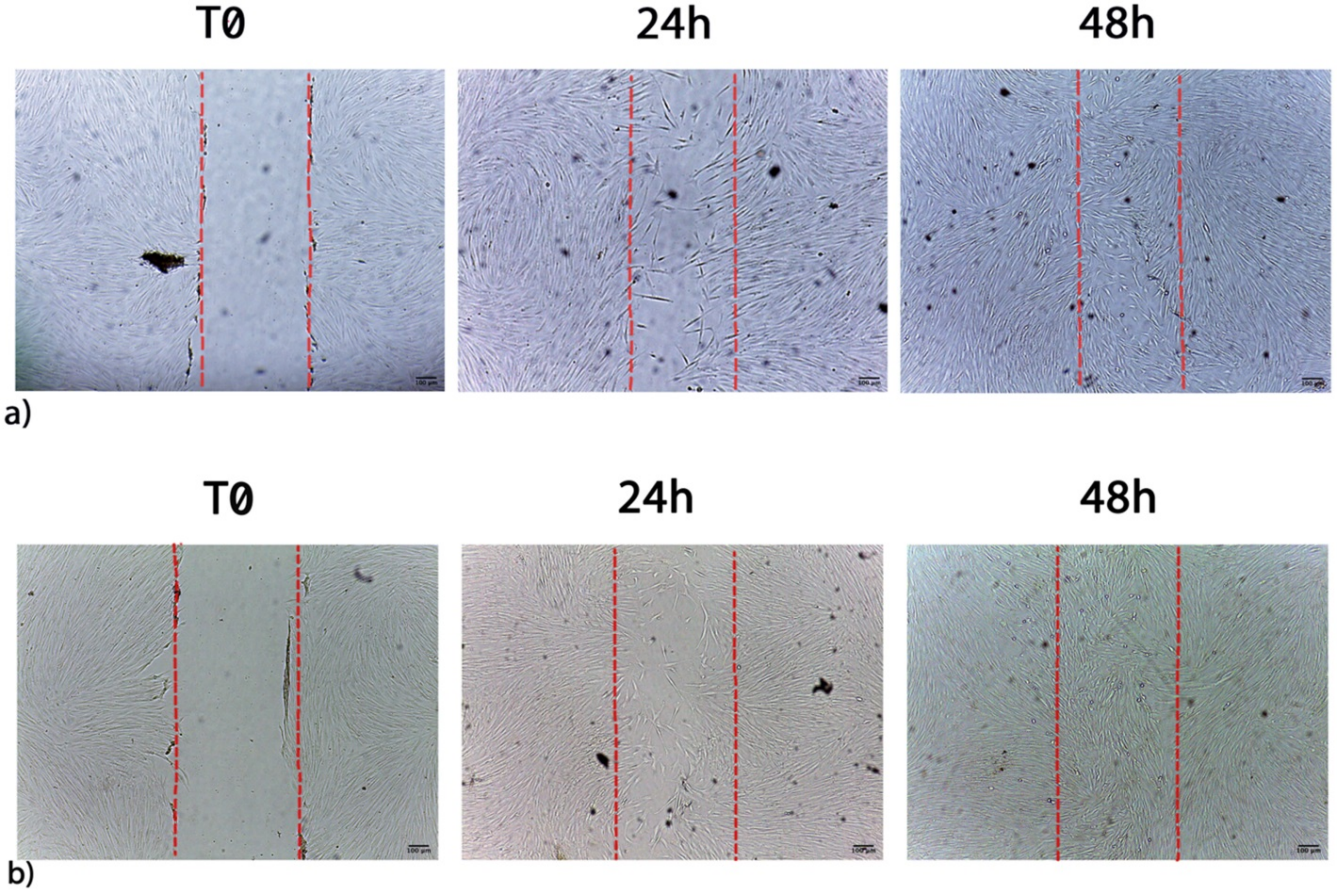
** a)** Migration of control cells after scratch. **b)** Migration of fibroblasts after scratch and treatment with 1 µL/mL L extracts. Images are acquired by optical microscope and are examples of different independent experiments. Scale bar = 100 µm.

The ability of waste extracts from leaves of C or L or H to stimulate fibroblast proliferation and/or migration was visualized by the scratch assay. Figure 5 shows the migration of fibroblasts after scratch and culture with 1 μL/mL L extract, being an example of the single extract effect on wound healing (C, L, H). L waste extract increased the number of cells detectable in the artificial wound site (Table 6) reaching the maximum stimulatory effect, after 72h of treatment (Table 6). When cells were cultured with 1 μL/mL of C or H waste extracts a lower migration and proliferation activity was observed, as compared to L-treated cells (Table 6).

The migration and proliferation of fibroblasts was stimulated as early as 24h by waste extracts of L or C as compared to control untreated cells, with a wound closure of 21,3%, and 21,7% respectively when a concentration of 1 μL/mL was applied.

Wound closure was also stimulated after 48 and 72 hours of culturing with low concentration (1 μL/mL) of both L (27.4% and 29.2%) or C (26.1% and 27.2%), both being less effective at higher concentration (Table 6). A completely different trend was observed while testing H waste extract, able to stimulate cell proliferation and migration only at 5 μL/mL.

**Figure 6:**
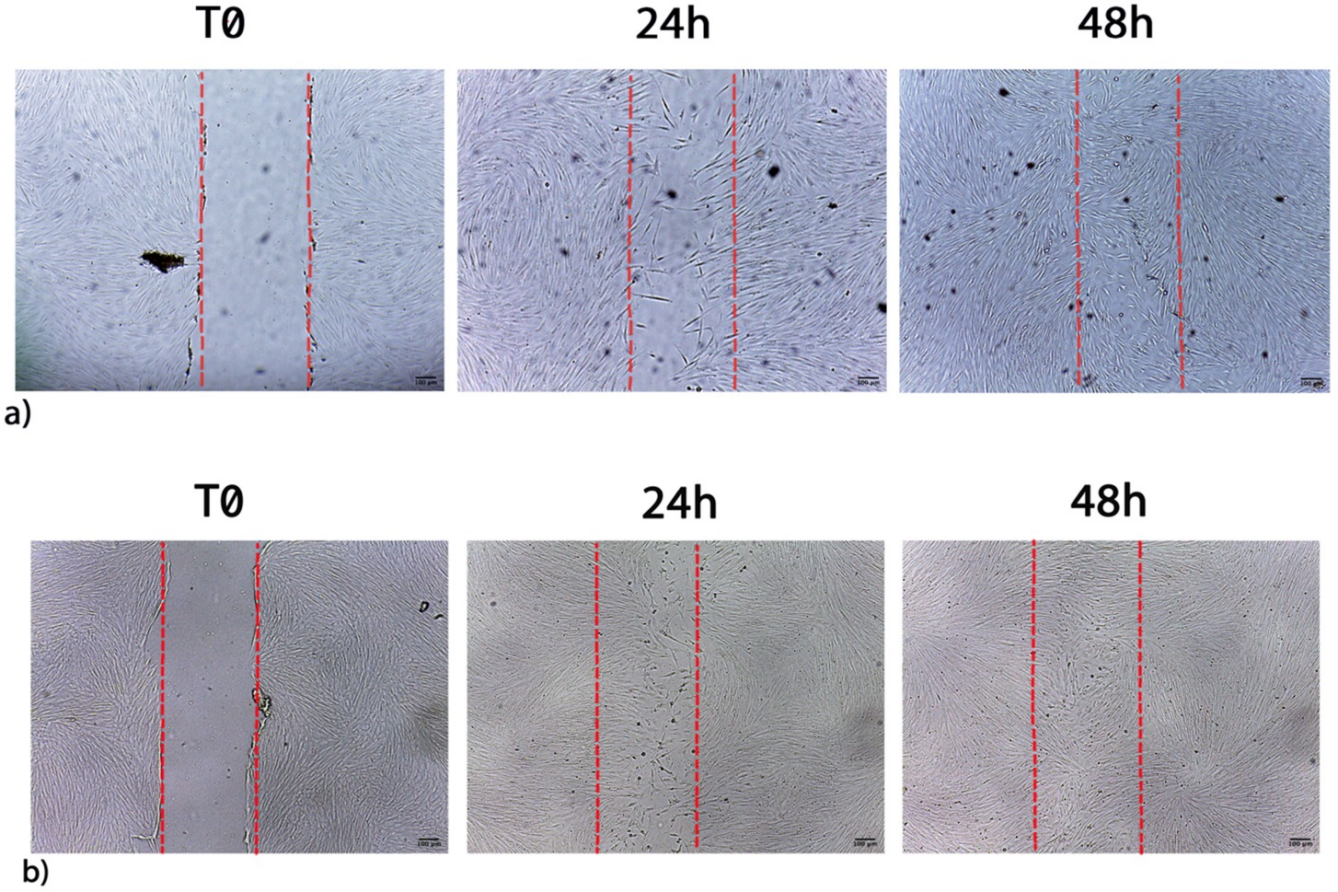
** a)** Migration of control cells after scratch. **b)** Migration of fibroblasts after scratch and treatment with 1 μL/mL CLH extracts. Images are acquired by optical microscope and are examples of different independent experiments. Scale bar = 100 µm.

Figure 6 shows the migration of fibroblasts after scratch and culture with 1 μL/mL CLH extracts, being examples of the pooled waste extracts effect on wound healing (CLH, CL, CH, LH). Table 7 shows the effect of pooled waste extracts (CLH, CL, CH, LH) on fibroblasts migration and proliferation, as percentages of wound closure.

Cells cultured with 1 μL/mL CH or CLH exhibited a complete wound closure within 48h, earlier than in control untreated cells. The wound healing stimulation was also observed when higher concentration of CH waste extracts (5 and 10 μL/mL) were added to cells for 72 h (Table 7).

Furthermore, 1 μL/mL LH waste extracts elicited an effect on wound healing starting from 48 h.

Wound closure time was faster in cells exposed to the combination of extracts as compared to cells exposed to one single extract. Indeed a greater cell proliferation and wound closure can be observed after 24h in cells exposed to the combination of extracts (CLH) (Figure 6, panel b).

**Figure 7:**
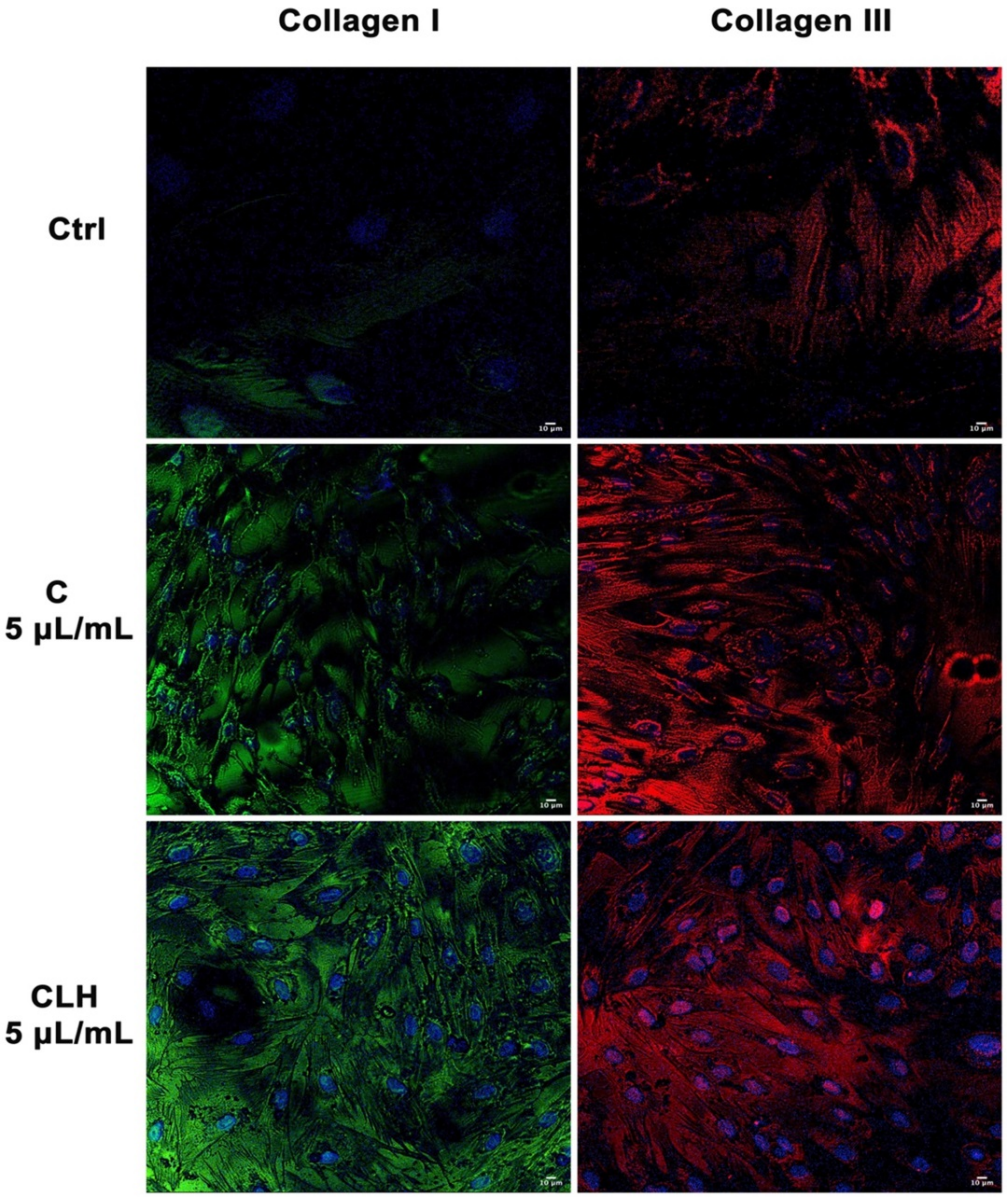
Analysis of collagen deposition during wound healing. Immunohistochemical analysis of the expression of Collagen type I and type III was assessed in fibroblasts after scratch and treatment with different concentrations of single waste extract (C, L, H) or pooled waste extracts (CLH, CL, CH, LH) for 72 hours. Control cells were cultured in basic growing medium. Nuclei are labelled with 4,6-diamidino-2-phenylindole (DAPI, blue). Scale bars: 10 µm. The figures are representative of different independent experiments. For each differentiation marker, fields with the highest yield of positively stained cells are shown.

Figure 7 shows the expression of collagen I and III in cells exposed to single waste extracts or pooled waste extracts, as compared to control untreated cells. Collagen deposition was higher in cells treated with the extracts than control cells, indicating a more efficient and rapid wound closure in the presence of the tested extracts.

## Discussion

Tissue regeneration is a quite complex process that consists in a sequence of cellular events leading to restore functionality. This reparative event involves cell migration, the deposition of collagen and the secretion of soluble mediators 35. Fibroblasts play a crucial role in skin wound closure, from the early inflammatory phase to the final extracellular matrix production that is essential to restore physical barrier 36, 37. Actually, plants and herbs, as active medicinal, are applied to stimulate stem cell proliferation, regeneration and rehabilitation in damaged tissues, being nowadays still employed in the attempt to counteract different groups of diseases 38. Moreover, different natural compounds, as bioactive molecules are largely used *in vitro* to condition specific culturing media in the attempt to activate stem cell regenerative potential 39-42. On the other hand, they have been also largely exploited in cancer treatment, for their capability to inhibit cell proliferation and neoplastic progression 43-46. The use of plant extracts or plant-derived compounds are largely preferred because of their widespread availability, fewer side effects, and effectiveness as crude preparations 47,21. Additionally, new green practices employ waste materials, after essential oil production from aromatic plants, with zero, or low-cost raw material prices. These waste-derived plant phenolics could be used as antioxidants in food products, pharmaceuticals, for a more sustainable approach on the whole plant 48, 49. Moreover, the management of cutaneous wounds can be further elicited by the associated antioxidant activity of some plant extracts 22, 50, 51. Components of traditional medicinal plants, as flavonoids, saponins, phenols, tannins and essential oils, are known for their antiseptic, anti-inflammatory, antioxidant properties, collagen synthesis stimulation and cell proliferative activities, biological processes deeply involved in wound healing 52. Therefore phytochemical screening assay which is considered a simple, quick, and inexpensive procedure 53, was the basis of our studies. Total phenolic amounts, were significantly higher in waste extracts from L and H if measured up to C. Phenolic compounds are well-known molecules that have many biological properties, including antioxidant activity, that play an important role in wound healing by preventing and protecting oxidative damage from free radicals 54. L and also H extracts exhibited a strong antioxidant activity, significantly higher (P<0.01) as compared to the effect elicited by C, along all the experimental time points of the ABTS assay (0-50 minutes, Figure 2 panel a). The positive Pearson correlation between antioxidant activity measured by ABTS and total phenolic contents, indicate that the antioxidant activity depends on the amount of the phenolic fraction, rosmarinic acid (L) and caffeoylquinic acid derivatives (H and C), confirming what previously showed by other authors 55-58. Moreover, even though the amount of total phenolic amounts was similar in all the pooled wasted extracts (CL, CH, LH, CLH), LH waste extracts showed a better antioxidant activity, similar to the positive control (Trolox). We can hypothesize that this different effect could be explained by a different concentration of various compounds, as for example saponines, tannins, alkaloids, alkenyl phenols, glycoalkaloids, flavonoids, sesquiterpenes lactones, terpenoids and phorbol esters, previously described by other authors 59. Interestingly, caffeoylquinic acid derivatives were the abundant phenol present in all the waste extracts except for CL in which rosmarinic acid was predominant. In LH the amount of caffeoylquinic acid derivatives was followed by rosmarinic acid, acting in a synergic manner to enhance the biological activity of the extracts 56, while in CL the antioxidant activity was mainly related to the presence of rosmarinic acid and rosmarinic acid derivatives. Among them some were previously described able to act synergistically by enhancing the bioactivity of each single compound 60. Our results perfectly fit with the existing literature, which underlined the relationship between antioxidant activity, the presence of phenolic compounds and cell proliferation 52. Nevertheless, our results highlight a further use of waste materials to ameliorate regenerative and antioxidant processes. Actually some authors described that extracts from L 61 and C 62 exert a high inhibitory effect on cancer cell proliferation *in vitro*, without affecting healthy normal cells growing at low concentrations 45, showing a dual and opposite effect depending on the concentration and time of exposure to the plant extracts. At the same time, our waste extracts didn't exhibit any cytotoxic effects on cell proliferation, especially at low concentrations and for prolonged culturing time, both alone (C, L, H) or as pooled waste extracts (CL, CH, LH, CLH). Our results confirm previous studies on aqueous extracts of the same plants, validating that waste material is still rich in bioactive compounds, 63. In the present paper we describe for the first time stimulatory effects of waste extracts from leaves of L, or H. or C on fibroblast migration. Fibroblasts are known to play an essential role in wound healing, and cell migration is one of the most essential steps, during the proliferative phase, responsible for wound closure 1, 21, 64, 65. Moreover, fibroblasts are involved in extracellular matrix synthesis, together with secretion of different growth factors as transforming growth factor (TGF-β1) and basic fibroblast growth factor (b-FGF), able to induce hyaluronic acid secretion and matrix deposition 66. Here we demonstrate that our extracts are able to induce Collagen I and III deposition during wound healing, thus accelerating the repairing process (Figure 7). The stimulation of fibroblast migration and proliferation, elicited by L and C waste extracts alone or the combination of CH and CLH pooled waste extracts; demonstrate that these compounds could contribute in the rebuilding of new granulation tissue thus restoring normal skin function. Our data further infer the use of post-distillation waste extracts to induce tissue remodeling stimulating anti-inflammatory responses and wound healing, as the aqueous extracts previously described by other authors 8,67. Within this context, the waste extracts described here could be used in the future in different combination, to stimulate wound healing processes and antioxidant responses in damaged skin, thanks to their synergistic effect.

## Conclusions

The data described in our study unravel a novel role of *L. stoechas, H. italicum.* and *C. arvensis* leaves waste extracts, and pooled combination of them, for the treatment of skin wounds. Taken together, our preliminary results demonstrate that waste extracts exhibit *in vitro* regenerative properties after environmental stress exposure and antioxidant activities related to the presence of phenolic compounds. Further *in vitro* and *in vivo* studies are needed to translate these results in future applications for tissue regeneration.

## Figures and Tables

**Table 1:** Determination of phenols by Folin-Ciocalteau's method.

(µL)	C µg GAE	H µg GAE	L µg GAE
25.00	384.08 ± 37.58	663.11 ± 42.71*	819.62 ± 57.20*
10.00	164.56 ± 6.61	378.05 ± 37.34	612.85 ± 48.88
5.00	90.89 ± 7.15	199.36 ± 20.78	426.90 ± 140.77
1.00	34.43 ± 4.63	49.55 ± 7.40	98.70 ± 21.38

Data were expressed as mean ± SD of 3 independent experiments and indicated as µg of gallic acid equivalent (GAE). A *P<0.05 was considered significant. *C. arvensis =* (C),* H. italicum* = (H),* L. stoechas =* (L).

**Table 2:** Phenolic compounds quantified in C, L and H decoction water.

Phenolic compounds	C mg/L ± DS	H mg/L ± DS	L mg/L ± DS
Caffeoylquinic acid derivatives^(£)^	112.21 ± 6.22	379.76 ± 11.25	77.77 ± 5.20
Quercetin glycoside derivatives^(&)^	70.84 ± 5.14	-	-
Luteolina derivatives^(ç)^	-	-	54.49 ± 5.02
Rosmarinic acid	-	-	382.46 ± 15.02
Rosmarinic acid derivatives^($)^	-	-	249.80 ± 8.15
Apigenin derivatives^(§)^	-	-	18.40 ± 1.55
Naringenin derivatives^(ø)^	-	24.02 ± 0.87	-

Results are expressed as mean ± standard deviation of 3 determinations. (£) Caffeoylquinic acid derivatives were expressed as chlorogenic acid. (&) Quercetin glycoside derivatives were expressed as quercetin. (ç) Luteolin glycoside derivatives were expressed as luteolin. ($) Rosmarinic acid derivatives were expressed as rosmarinic acid. (§) Apigenin glycoside derivatives were expressed as apigenin. (**ø**) Naringenin glycoside derivatives were expressed as naringenin.

**Table 3:** Determination of phenols by Folin-Ciocalteau's method of pooled waste extracts.

(µL)	CLH µg GAE	CL µg GAE	CH µg GAE	LH µg GAE
25.00	693.88 ± 5.01*	701.77 ± 19.22*	524.48 ± 65.77	697.73 ± 27.33*
10.00	471.66 ± 189.91	465.15 ± 133.34	241.20 ± 106.28	495.22 ± 87.77
5.00	240.77 ± 74.10	265.30 ± 79.93	162.58 ± 54.77	387.77 ± 213.59
1.00	72.61 ± 36.75	75.26 ± 31.80	46.89 ± 6.48	106.72 ± 25.10

Data were expressed as mean ± SD of 3 independent experiments and indicated as µg of gallic acid equivalent (GAE). A *P<0.05 was considered significant. *C. arvensis / L. stoechas / H. italicum* = (CLH), *C. arvensis / L. stoechas* = (CL), *C. arvensis / H. italicum* = (CH), *L. stoechas / H. italicum* = (LH).

**Table 4:** Phenolic compounds quantified in CLH, CL, CH and LH decoction water.

Phenolic compounds	CLH mg/L ± DS	CL mg/L ± DS	CH mg/L ± DS	LH mg/L ± DS
Caffeoylquinic acid derivatives^(£)^	54.84 ± 2.29	65.62 ± 3.22	262.18 ± 9.51	207.85 ± 7.51
Luteolin derivatives^(&)^	21.84 ± 0.87	45.45 ± 1.76	-	35.60 ± 2.00
Rosmarinic acid	29.20 ± 1.59	160.02 ± 8.98	-	81.95 ± 4.02
Rosmarinic acid derivatives^($)^	-	79.86 ± 4.15	-	29.12 ± 1.49
Quercetin glycoside derivatives^(ç)^	28.68 ± 0.98	40.30 ± 1.05	28.98 ± 1.09	-
Apigenin derivatives^(§)^	17.98 ± 1.02	-	-	5.68 ± 0.08
Neoeriocitrin derivative^(ø)^	41.92 ± 1.54	-	26.49 ± 1.05	14.93 ± 0.29

Results are expressed as mean ± standard deviation of 3 determinations. (£) Caffeoylquinic acid derivatives were expressed as chlorogenic acid. (&) Luteolin glycoside derivatives were expressed as luteolin. ($) Rosmarinic acid derivatives were expressed as rosmarinic acid. (ç)Quercetin glycoside derivatives were expressed as quercetin. (§) Apigenin glycoside derivatives were expressed as apigenin. (ø)Neoeriocitrin glycoside derivatives were expressed as neoeriocitrin.

**Table 5:** Spearman correlation between absolute value of antioxidant activity and phenols amounts

(µL)	ABTS C	Phenols C	ABTS L	Phenols L	ABTS H	Phenols H	Spearman correlation
25,00	107,25	384,08	112,34	819,62	108,12	663,11	P<0.05
10,00	103,87	164,56	110,13	612,85	107,93	378,05	
5,00	93,88	90,89	108,34	426,90	105,24	199,36	
1,00	52,43	34,43	105,98	98,70	87,11	49,55	

*C. arvensis* = (C), *L. stoechas* = (L), and *H. italicum* = (H).

**Table 6:** Migration and proliferation of fibroblasts stimulated by waste extracts

hours	Untreated	C				L				H		
		1 μL/mL	5 μL/mL	10 μL/mL		1 μL/mL	5 μL/mL	10 μL/mL		1 μL/mL	5 μL/mL	10 μL/mL
24	16.2±2.5	21.3±1.68	20.3±2.9	20.1±2.5		21.7±3.0	16.7±1.3	14.1±3.2		14.2±3.4	17.1±3.0	14.4±1.9
48	20.4±0.4	26.1±0.8	22.8±0.4	23.1±0.6		27.4±1.9	21.7±1.7	21.0±3.6		19.3±0.2	22.8±0.3	16.0±0.0
72	21.0±0.0	**27.2±0.0**	**23.8±0.0**	**24.1±0.0**		**29.2±0.5**	**21.1±0.6**	**23.85±0.6**		**19.9±0.0**	**23.6±0.0**	**16.4±0.0**

Data were expressed as wound closure percentage (%) at different time points (24, 48, 72 hours) untreated and stimulated by *C. arvensis* = (C), *L. stoechas =* (L)*,* and* H. italicum* = (H), at different concentration (1, 5, 10 μL/mL).

**Table 7:** Migration and proliferation of fibroblasts stimulated by pooled waste extracts

hours	Untreated	CLH			CL			CH			LH		
		1 μL/mL	5 μL/mL	10 μL/mL	1 μL/mL	5 μL/mL	10 μL/mL	1 μL/mL	5 μL/mL	10 μL/mL	1 μL/mL	5 μL/mL	10 μL/mL
24	24.9±1.8	27.6±1.6	21.7±2.7	19.5±4.2	24.2±3.8	18.4±3.1	14.1±1.9	23.8±2.0	20.8±2.4	17.6±2.4	24.7±1.3	20.8±2.4	18.6±1.5
48	30.6±2.1	**31.0±0.0**	29.2±0.9	27.2±2.5	29.8±0.8	27.4±1.9	22.6±2.2	**30.6±0.0**	**32.1±0.6**	**30.0±1.5**	30.9±0.7	28.0±0.9	24.2±1.9
72	31.8±0.0	**closed**	31.2±0.0	32.3±1.7	30.4±0.0	30.1±0.2	28.0±1.6	**closed**	**33.0±0.0**	**32.6±0.0**	31.7±0.0	30.2±0.2	28.7±1.1

Data were expressed as wound closure percentage (%) at different time points (24, 48, 72 hours) untreated and stimulated by pooled waste extracts of *C. arvensis*,* L. stoechas* and *H. italicum* (CLH, CL, CH, LH) at different concentration (1, 5, 10 μL/mL).
